# In this issue: Volume 116, Issue 1, January 2025

**DOI:** 10.1111/cas.16435

**Published:** 2025-01-08

## SET facilitates immune escape of microsatellite stability colorectal cancer by inhibiting c‐Myc degradation

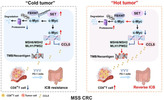

Colorectal cancer (CRC) is a common type of cancer that affects many people worldwide. Most CRC cases are classified as microsatellite stable (MSS) CRC, which are particularly challenging to treat. A promising treatment called immune checkpoint blockade (ICB) therapy helps the immune system fight cancer, but MSS CRC doesn't respond well to it. This is because MSS CRC lacks key features, such as high mutation rates and active immune cells, that make it harder for the immune system to recognize the tumor as a threat.

Researchers have identified a potential culprit behind this—the SET protein. SET is found in higher amounts in MSS CRC compared to another type of CRC that responds better to ICB therapy. SET increases the activity of mismatch repair proteins, which reduce mutation rates and prevent the immune system from recognizing the tumor. SET also suppresses the production of CCL5, a signaling molecule that normally attracts immune cells to the tumor, enabling the tumor to grow unchecked. Additionally, SET stabilizes c‐Myc, a protein that promotes tumor growth and prevents immune cells from infiltrating it.

By blocking SET, researchers made the tumors more visible to the immune system. As a result, more immune cells could infiltrate the tumor, making the cancer more susceptible to treatment. When SET inhibition was combined with ICB therapy, the immune response against MSS CRC was significantly improved.

These findings suggest that targeting SET could be a new way to make MSS CRC more responsive to immunotherapy, improving treatment outcomes for patients with this difficult‐to‐treat cancer.

https://onlinelibrary.wiley.com/doi/10.1111/cas.16368

## Noncanonical TCA cycle fosters canonical TCA cycle and mitochondrial integrity in acute myeloid leukemia

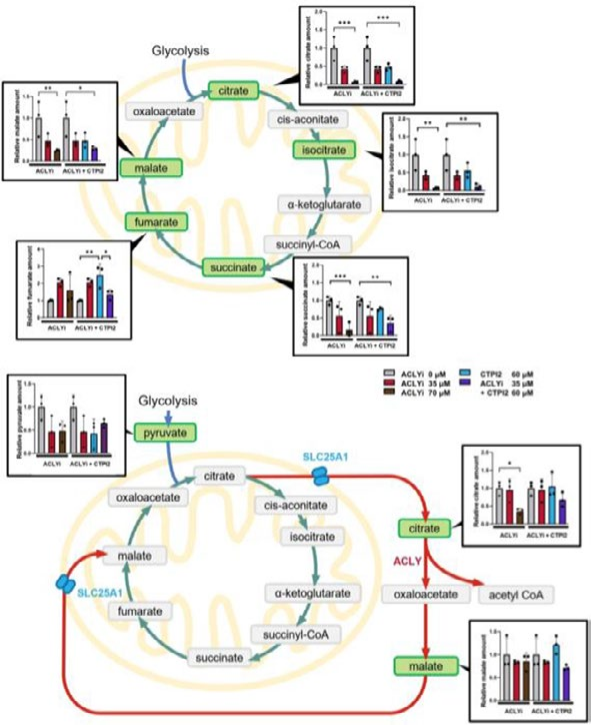

The tricarboxylic acid (TCA) cycle, also known as the Krebs cycle, is a fundamental process within mitochondria that metabolizes nutrients to produce ATP, the primary energy currency of cells. While the canonical TCA cycle involves eight well‐known mitochondrial reactions, recent studies have identified a non‐canonical TCA cycle that partially takes place in the cytosol. Cancer cells often reprogram their metabolism to utilize these cycles for growth and survival. Although the canonical TCA cycle has been widely studied in cancer biology, the non‐canonical TCA cycle and its interactions remain less understood.

A central enzyme in the non‐canonical TCA cycle is ATP‐citrate lyase (ACLY), which converts citrate to acetyl‐CoA. Overexpression of ACLY is common in many cancers, and studies have shown that its inhibition can have anti‐tumor effects, underscoring the importance of the non‐canonical TCA cycle in cancer progression and its interaction with the canonical TCA cycle.

This study explored metabolic associations between the non‐canonical and canonical TCA cycles in acute myeloid leukemia, a type of blood cancer, and the role of ACLY in supporting mitochondrial function in the cancer cells.

The results showed that inhibiting ACLY in cancer cells increased reactive oxygen species (ROS), harmful byproducts of oxidative phosphorylation (OXPHOS). This led to DNA damage, reduced mitochondrial membrane potential, and induced apoptosis. These effects were further amplified by CTPI2, a mitochondrial citrate transporter inhibitor, which disrupted ATP production and reduced ACLY activity.

Using the ATP Tracking Harmonized ENergy‐shift Assay (ATHENA), the study demonstrated that ACLY inhibition lowered citrate levels in the cytosol. It also shifted the TCA cycle more towards OXPHOS, increasing ROS generation. Meanwhile, CTPI2 lowered ACLY activity. This suggested at the non‐canonical TCA cycle being sustained by a positive feedback loop, where citrate levels and ACLY activity were interdependent. Metabolomic analysis indicated that ACLY inhibition, with or without CTPI2, reduced the levels of several canonical TCA cycle metabolites in mitochondria, with fumarate being the exception. These findings highlight the critical role of the non‐canonical TCA cycle in sustaining the canonical TCA cycle and mitochondrial integrity.

In conclusion, the non‐canonical TCA cycle is indispensable for mitochondrial function and energy production in cancer cells. Targeting ACLY and mitochondrial citrate transporters presents a promising therapeutic strategy to impair cancer metabolism and hinder tumor growth.

https://onlinelibrary.wiley.com/doi/10.1111/cas.16347

## Evaluating stiffness of gastric wall using laser resonance frequency analysis for gastric cancer

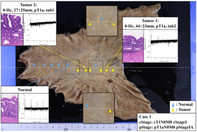

Detecting stomach cancer early is crucial because it significantly improves treatment outcomes. However, current diagnostic methods, like endoscopic imaging, often rely on the skill of the operator and can struggle with certain types of tumors, especially those that don't show clear changes on the surface.

This study focuses on an innovative approach called Laser Resonance Frequency Analysis (L‐RFA), which measures the stiffness of tissues. Tumors are generally stiffer than normal tissue, and this difference could help doctors pinpoint cancerous areas more effectively. Using lasers to study tissue stiffness might sound like science fiction, but L‐RFA works without direct contact with the tissue, making it non‐invasive and potentially more comfortable for patients.

The researchers tested this technology on samples from two patients with early‐stage stomach cancer who underwent surgery. By comparing the vibrations of tumor and healthy areas, they found clear distinctions. Tumor areas showed weaker vibrations, confirming that they are stiffer than normal tissue. These findings align with earlier tests using artificial tissue models, which also demonstrated that L‐RFA accurately measures stiffness.

While promising, this study has had limitations, including a small sample size and potential influence of tissue inflammation. More research is needed to confirm L‐RFA's performance in real‐world conditions and during live procedures. Despite challenges, L‐RFA's potential is significant. It could complement existing techniques, enabling more precise treatment decisions. This innovation represents a step toward smarter, more accurate cancer diagnostics.

https://onlinelibrary.wiley.com/doi/10.1111/cas.16383

